# D1-plus vs D2 nodal dissection in gastric cancer: a propensity score matched comparison and review of published literature

**DOI:** 10.1186/s12893-020-00714-x

**Published:** 2020-06-10

**Authors:** Laura Lorenzon, Rosina Giudicissi, Marco Scatizzi, Genoveffa Balducci, Stefano Cantafio, Alberto Biondi, Roberto Persiani, Paolo Mercantini, Domenico D’Ugo

**Affiliations:** 1 General Surgery Unit, Fondazione Policlinico Universitario Agostino Gemelli, Catholic University, Largo Francesco Vito 1, 00168 Rome, Italy; 2 Department of General and Oncologic Surgery, Santo Stefano Hospital, Prato, Italy; 3 Surgical and Medical Department of Traslational Medicine, Sant’Andrea Hospital, Faculty of Medicine and Psychology, Sapienza University of Rome, via di Grottarossa 1035, Rome, 00185 Italy

**Keywords:** Gastric cancer, Nodal dissection, D1-plus, D2, Lymphadenectomy, PSM

## Abstract

**Background:**

The results of D1-plus lymphadenectomy following gastric resection are seldom investigated. The aim of this study was to compare results of D1-plus vs D2 resections and to provide a literature review.

**Methods:**

Patients who underwent upfront R0 gastrectomy for adenocarcinoma from 2000 to 2016 in three Institutions were selected using propensity scores and categorized according to lymphadenectomy. Statistical analyses were performed for the nodal harvest (LNH) and survival. Published literature comparing D1-plus and D2 was reviewed and analyzed according to PICO and PRISMA guidelines.

**Results:**

Two matched groups of 93 D1-plus and 93 D2 resections were selected. LNH was significantly greater in D2 vs D1-plus dissections (mean 31.2 vs 27.2, p 0.04), however LNH distribution was similar. The cumulative incidence curves for overall survival, disease free and disease specific events did not report significant differences, however Cox regression analysis disclosed that total gastrectomies (HR 1.8; 95% 1.0–2.9), advanced stages (HR 5.9; 95% 3.4–10.3) and D1-plus nodal dissection (HR 2.1; 95% 1.26–3.50) independently correlated with disease free survival. Literature review including 297 D1-plus and 556 D2 lymphadenectomies documented LNH in favor of D2 sub-group (SMD -0.772; 95%CI -1.222- -0.322).

**Conclusion:**

D2 provided greater LNH than D1-plus dissections; prospective studies should aim to investigate long-term survival of D1-plus lymphadenectomy.

## Background

Gastric cancer is still the fourth most common cause of cancer death [1]. Even if current international guidelines recommend a multimodal approach [2–5], the surgical resection remains an essential part of the multidisciplinary care. The surgical procedures for gastric cancers should aim to achieve a curative R0 resection and -according to the American Joint Committee on Cancer- an appropriate dissection of at least 15 nodes [6].

With respect of lymphadenectomy, the Japanese Research Society for Gastric Cancer described different techniques: D1; D1-plus -including D1 stations and 8a, 9 and 11p (total gastrectomy) or 8a and 9 (in distal resection)-; D2; D2 extended (so-called “D2+”) - currently recommended for metastases at the infra-pyloric nodes - and, finally, a super-extended (D3) dissection [7, 8].

More than 50 years ago, the Japanese Gastric Cancer Treatment guidelines claimed that D2 dissection was the gold standard [9]. However, in Western countries, results from large trials comparing D1 vs D2 lymphadenectomies documented contradictory results. Indeed, the 15-years follow-up analysis from the Dutch gastric cancer group, reported that D2 lymphadenectomy was associated with lower local recurrences, higher morbidity rates and improved survival only in N2 patients [10, 11], in line with the historical results of the Medical Research Council study [12].

On the other hand, D1-plus dissections could aim to achieve a sufficient harvest for an adequate staging. Nowadays, guidelines recommend D1-plus nodal dissection only in selected cases, like early gastric cancers not suitable for endoscopic resections, or T1 tumors [13–17], but the results of this approach are very seldom investigated, in particular in comparison with D2 lymphadenectomy [18], [19].

Lymph-node harvest is still a matter of importance; indeed, two international data-sets (US and Korea), analyzing more than 25.000 gastric cancer patients, recently highlighted a statistically significant survival improvement for those patients with more than 29 nodes analyzed [20].

This investigation was based on the hypothesis that D1-plus and D2 lymphadenectomies could provide significant differences in the nodal harvest and survival outcomes of gastric cancer patients.

On this basis, this study aimed to analyze a matched series of gastric cancer patients who underwent D1-plus lymph-node dissection and D2 lymphadenectomy, in relation to the lymph-node harvest (LNH) and long term survival. The secondary aim was to provide a literature review of updated studies in this field.

## Methods

### Study design

**Fig. 1:**
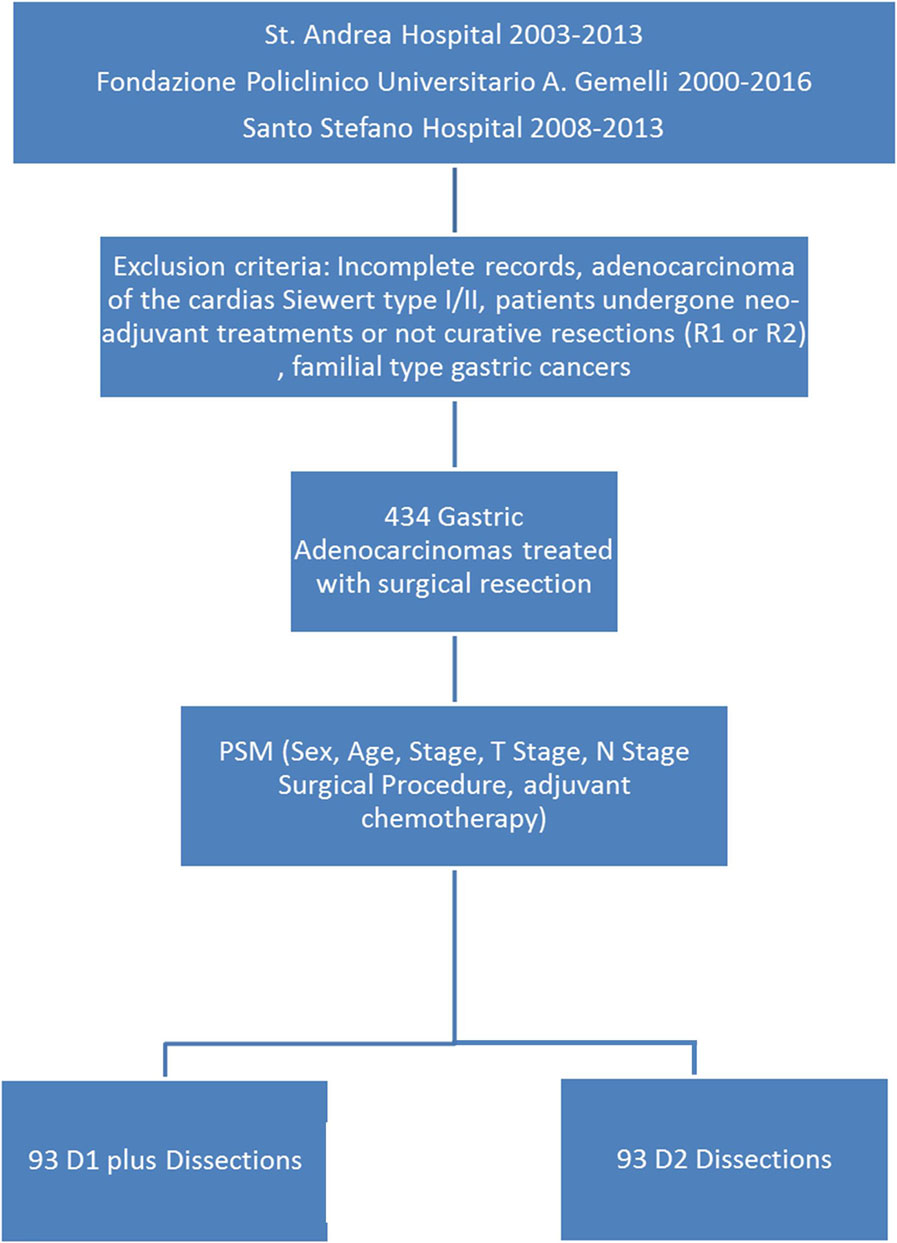
Clinical study design and propensity score method

Figure 1 outlines the study design. Clinical records were retrieved from internal databases of three Italian Institutions: Sant’Andrea University Hospital (Rome), Fondazione Policlinico Universitario A. Gemelli Hospital IRCCS (Rome) and Santo Stefano Hospital (Prato). All the consecutive patients who underwent elective R0 upfront resection for gastric adenocarcinoma from January 2000 to October 2016 were included. Patients from D1-plus group were all treated at the Sant’Andrea Hospital (where D1-plus dissection is the standard lymphadenectomy), whereas D2 procedures were performed at the other 2 Institutions (where D2 procedures are standard practice).

The exclusion criteria were: incomplete records (surgery, demographics, pathology, chemotherapy, follow-up), adenocarcinoma located at the cardias Siewert type I/II, patients who underwent pre-operative chemo-radiation, R1/R2 resections and patients with genetic syndromes including CDH1 mutations.

### Patients and records

All data and materials’ where obtained from prospectively maintained Departments’ database. All the records were de-identified and pooled in a common database using a consecutive number. An authorization of the IRB was not required for the retrospective observational investigation but a signed consent for surgical treatment and research purposes was obtained in compliance with privacy of personal data (http://old.iss.it/binary/publ/cont/15_44_web.pdf). The data retrieved included: tumor location, surgical procedure, patient’s age and sex, adjuvant treatments. The pathological records included also grading, histology according to Lauren classification, stage (American Joint Committee on Cancer Manual 7th Edition), lymph-node harvest (LNH) and number of metastatic lymph nodes. Lymph-node ratio (LNR) was calculated as the ratio between the number of metastatic lymph nodes and LNH [21]. Moreover, patients were categorized into those who underwent LNH ≥ 15 and with LNH < 15 harvest [6].

### Surgery

All three Institutions are certified as high volume centers for gastric cancer treatment by Italian National Health Authorities [22]. The surgical resections were categorized into sub-total and total gastrectomies: the nodal dissections were performed at each center according with the principles of the Japanese Gastric Cancer Association [8]. Briefly, for total gastrectomy, the lymph nodes stations dissected in D1-plus lymphadenectomy were stations from 1 to 7, 8a, 9 and 11p; D2 lymphadenectomy included D1-plus dissection and stations 10, 11d, and 12a; splenectomy was not routinely performed. For distal gastrectomy, the lymph nodes stations dissected in D1-plus lymphadenectomy were stations 1, 3, 4sb, 4d, 5, 6, 7, 8a and 9; D2 included D1-plus and stations 11p, and 12a [7].

### Outcome measures

Outcomes included survival and LNH. Follow-up was conducted by telephone interviews with the following endpoints: overall survival (OS, all causes of death), disease free survival (DFS, first recurrence of the disease), and disease specific survival (DSS, death due to gastric cancer).

### Literature review for meta-analysis

**Fig. 2:**
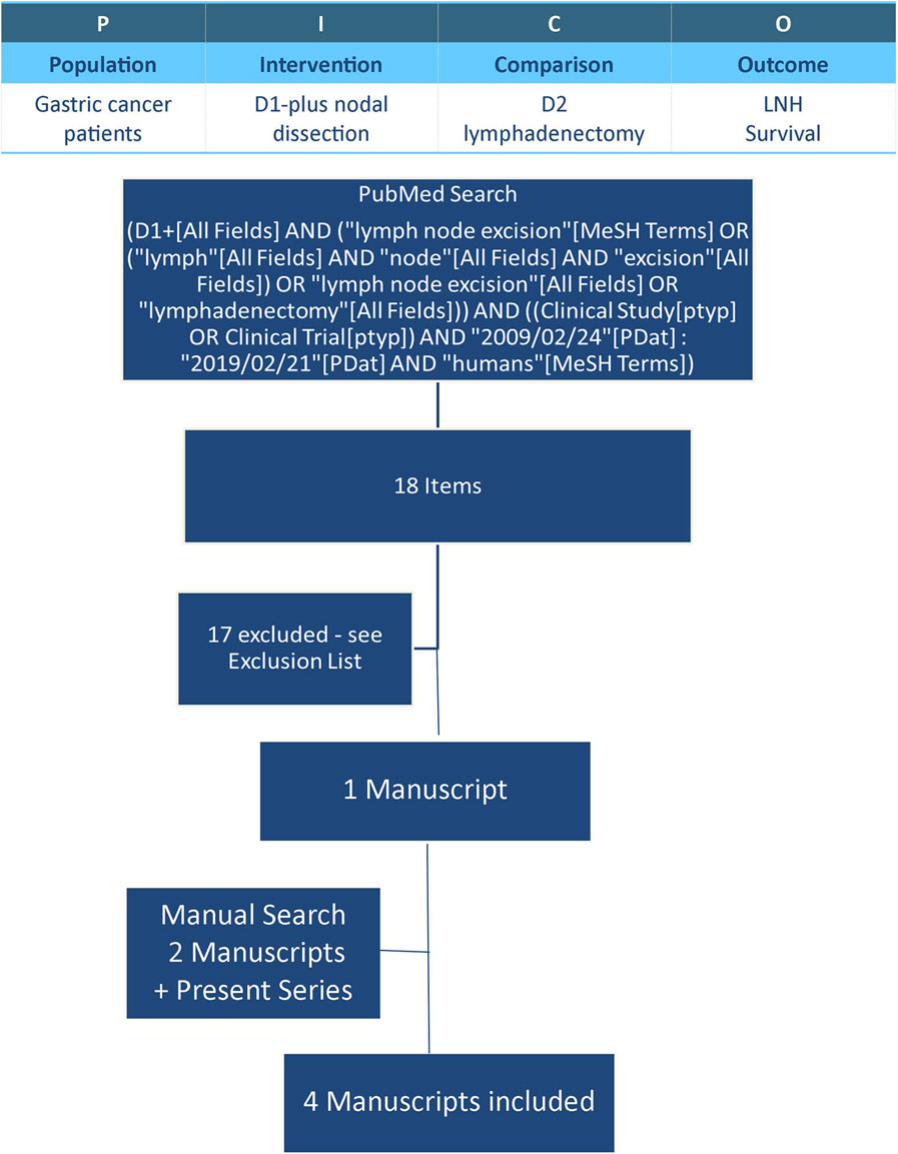
Meta-analysis design according to PRISMA guidelines for systematic review and meta-analysis

Review was conducted consistently with PICO questions and adhering to PRISMA statement, Fig. 2. A systematic review of the literature was conducted by searching PubMed database using the following search strategy: (D1 + [All Fields] AND (“lymph node excision”[MeSH Terms] OR (“lymph”[All Fields] AND “node”[All Fields] AND “excision”[All Fields]) OR “lymph node excision”[All Fields] OR “lymphadenectomy”[All Fields])) AND ((Clinical Study [ptyp] OR Clinical Trial [ptyp]) AND “2009/02/24”[PDat]: “2019/02/21”[PDat] AND “humans”[MeSH Terms]).

Authors of this study were blinded to authors’ and journals’ name while reviewing the series, and did not have any contacts with the authors of the included papers. Bibliometric indexes (e.g., journal’s Impact Factors) were not considered as an exclusion criteria. Main outcome measures were survivals and LNH.

Each paper retrieved was assessed for inclusion or exclusion by revision of the titles and the abstracts.

### Statistical analysis

Continuous variables were analyzed using means and standard deviations, whereas categorical variables were analyzed using frequencies and percent, and sub-groups were compared using the T-test and Chi-square tests. Propensity scores were calculated using a logistic regression model and the following covariates: age, sex, T-stage, N-stage, Tumor’s stage, surgical procedures (total/sub-total gastrectomy) and adjuvant chemotherapy treatment. D1-plus patients were then individually matched 1:1 to patients who underwent D2 dissection using the closer propensity score. LNH distributions were compared in the two sub-groups using the Kolmogorov-Smirnov test.

The cumulative incidence curves for OS, DFS and DSS events were calculated and sub-groups compared using the Grey test. Cox proportional-hazards regressions (forward method) were performed with the end-point of OS, DFS and DSS (co-variates: nodal dissection -D2 vs D1-plus-, surgical procedures -sub-total vs total gastrectomy-, age - < 65 yrs. vs ≥66 yrs-, Stage –Stage1–2 vs Stage 3–4, T-Stage – T1-T2 vs T3-T4, LNH 15 -LNH ≥ 15 vs LNH < 15, sex – male vs female).

For the literature review of studies with a continuous measure (comparison of means between treated cases and controls), the Hedges g statistic was used as a formulation for the standardized mean difference (SMD) under the fixed effects model. Next the heterogeneity statistic was incorporated to calculate the summary standardized mean difference under the random effects model. If the value 0 was not within the 95%CI, then the SMD is statistically significant at the 5% level (*P* <  0.05). Statistical heterogeneity of the results of the studies was assessed on the basis of a test of heterogeneity (standard chi-squared test on N degrees of freedom where N equals the number of trials contributing data minus one). If the test of heterogeneity was statistically significant (*p* <  0.05) then more emphasis should be placed on the random effects model.

All the tests performed two-tailed and a *p* value < 0.05 was considered as statistically significant. Statistical analyses were obtained using MedCalc for Windows, version 10.2.0.0, SPSS version 21.0 and XLSTAT 2019.3.2. A post hoc analysis was performed for evaluating the power of the tests using G*Power software version 3.1.2, with an effect size of 0.3.

## Results

### Patients

Four-hundred and thirty-four patients met the inclusion criteria. Using PSM two matched cohorts of 93 patients were selected, Fig. 1. Table 1 outlines the clinical features of the two sub-groups, documenting also the adequateness of the matching (Supplement Figure 1). As reported in Table 1, all analyses were documented with an adequate power 1-β > 0.80.

**Table 1:** Clinical, pathological and surgical features of D1-plus and D2 matched cohorts

	D1-plus		D2		*P* value	DF	Power 1-Beta^a^
	n	%	n	%			
Sex							
Female	40.0	43.0	39.0	43.0			
Male	53.0	57.0	54.0	57.0			
Total	93.0	100.0	93.0	100.0	1	1	0.98
Age (years)							
Mean; SD	67.8	11.6	65.5	13.0			
Median	69.0		68.0				
Range	31.0	87.0	29.0	88.0	0.20		0.98
Procedure							
Sub-total Gastrectomies	61.0	65.6	67.0	65.6			
Total Gastrectomies	32.0	34.4	26.0	34.4			
Total	93.0	100.0	93.0	100.0	0.42	1	0.98
T Stage	**n**	**%**	**n**	**%**			
T1 -T2	56.0	60.2	49.0	60.2			
T3-T4	37.0	39.8	44.0	39.8			
Total	93.0	100.0	93.0	100.0	0.37	1	0.98
N Stage							
N0	34.0	36.6	32.0	36.6			
N positive	59.0	63.4	61.0	63.4			
Total	93.0	100.0	93.0	100.0	0.87	1	0.98
LNH							
Mean; SD	27.2	12.5	31.2	13.9			
Median	26.0		29.0				
Range	2.0	71.0	1.0	64.0	**0.04**		0.98
N Positivity							
Mean; SD	5.7	9.3	4.9	7.8			
Median	2.0		1.0				
Range	0.0	48.0	0.0	40.0	0.53		0.98
LNH < 15							
LNH < 15	13.0	14.0	7.0	7.5			
LNH ≥ 15	80.0	86.0	86.0	92.5			
Total	93.0	100.0	93.0	100.0	0.22	1	0.98
LNR							
LNR0	34.0	36.6	32.0	36.6			
LNR1	9.0	9.7	15.0	9.7			
LNR2	8.0	8.6	10.0	8.6			
LNR3	13.0	14.0	10.0	14.0			
LNR4	8.0	8.6	6.0	8.6			
LNR5	21.0	22.6	20.0	22.6			
Total	93.0	100.0	93.0	100.0	0.77	5	0.9
Stage							
Stage 1–2	61.0	65.6	52.0	65.6			
Stage 3–4	32.0	34.4	41.0	34.4			
Total	93.0	100.0	93.0	100.0	0.22	1	0.98
Grading							
G1-G2	26.0	28.6	32.0	38.1			
G3-G4	65.0	71.4	52.0	61.9			
Total	91.0	100.0	84.0	100.0	0.23	1	0.97
Lauren Classification							
Intestinal	49.0	73.1	51.0	66.2			
Diffuse	18.0	26.9	26.0	33.8			
Total	67.0	100.0	77.0	100.0	0.47	1	0.94
Adjuvant Chemotherapy							
Performed	47.0	50.5	53.0	50.5			
Not Performed	46.0	49.5	40.0	49.5			
Total	93.0	100.0	93.0	100.0	0.46	1	0.98
Relapses							
Yes	34.0	36.6	33.0	36.6			
No	59.0	63.4	60.0	63.4			
Total	93.0	100.0	93.0	100.0	1	1	
Follow up (months)							
Mean; SD	36.8	26.4	83.5	68.9			
Median	32.5		63.0		**< 0.0001**		0.98
Range	3.0	100.0	3.0	267.0			

^a^effect size 0.03

### Nodal outcome

**Fig. 3:**
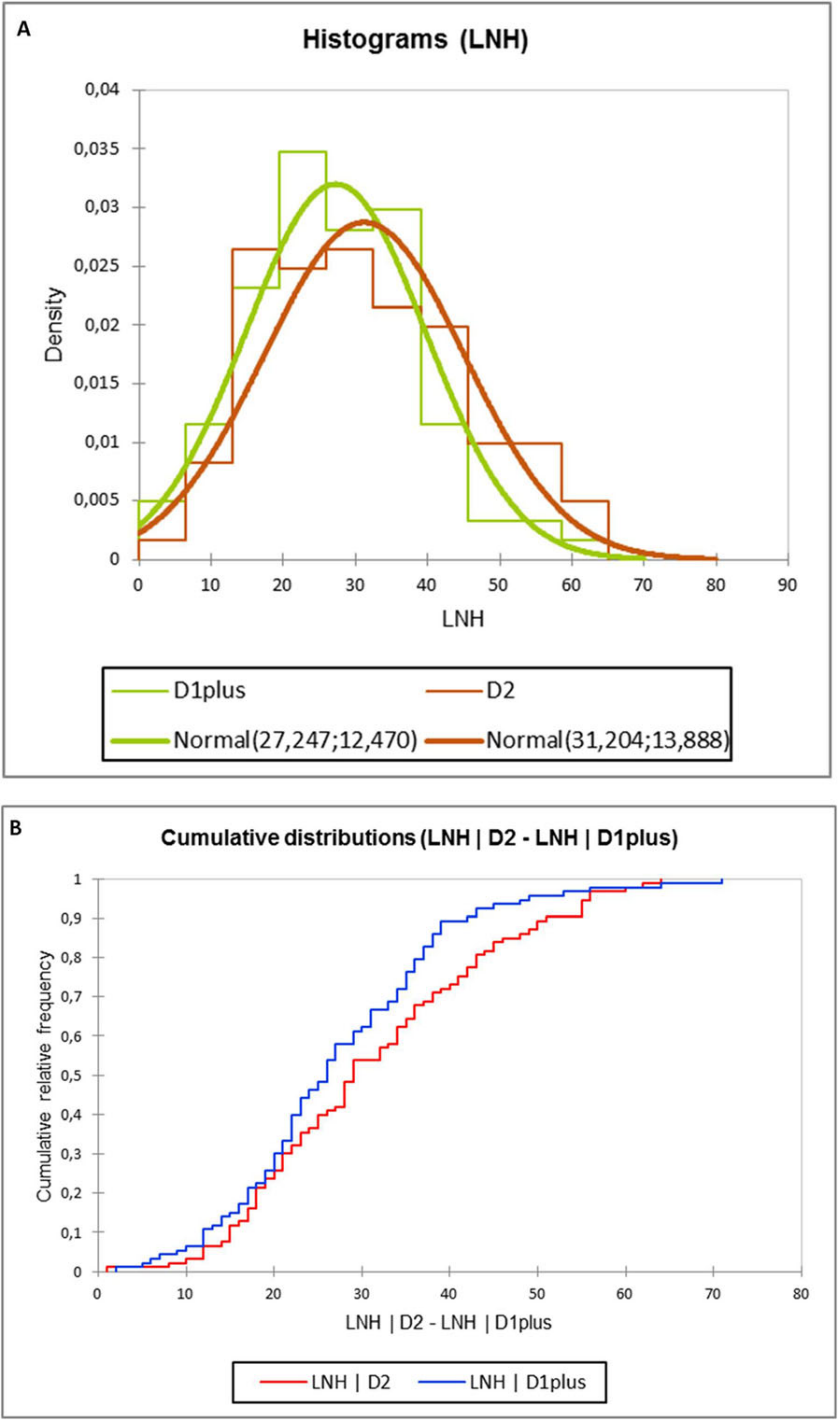
LNH distribution in D1-plus and D2 nodal dissections; **a** Histograms and Normal curves; **b** cumulative distributions in the two cohorts

Table 1 shows also the results of the comparison between the sub-groups with respect of nodal positivity and harvesting. As documented, D2 patients presented a significant greater harvest comparing D1-plus (mean LNH 31.2 vs 27.2, p 0.04). The patients who had LNH < 15 were 13 in the D1-plus sub-group, and 6 in the D2 group, however this difference was not reported as significant (p = NS). Figure 3 reports the LNH distribution in the two sub-groups: as the computed *p*-value of the Kolmogorov-Smirnov test was 0.127 and thus greater than the significance level alpha = 0,05, the null hypothesis H0 “the two samples follow the same distribution” was not rejected.

### Survival analysis

**Fig. 4:**
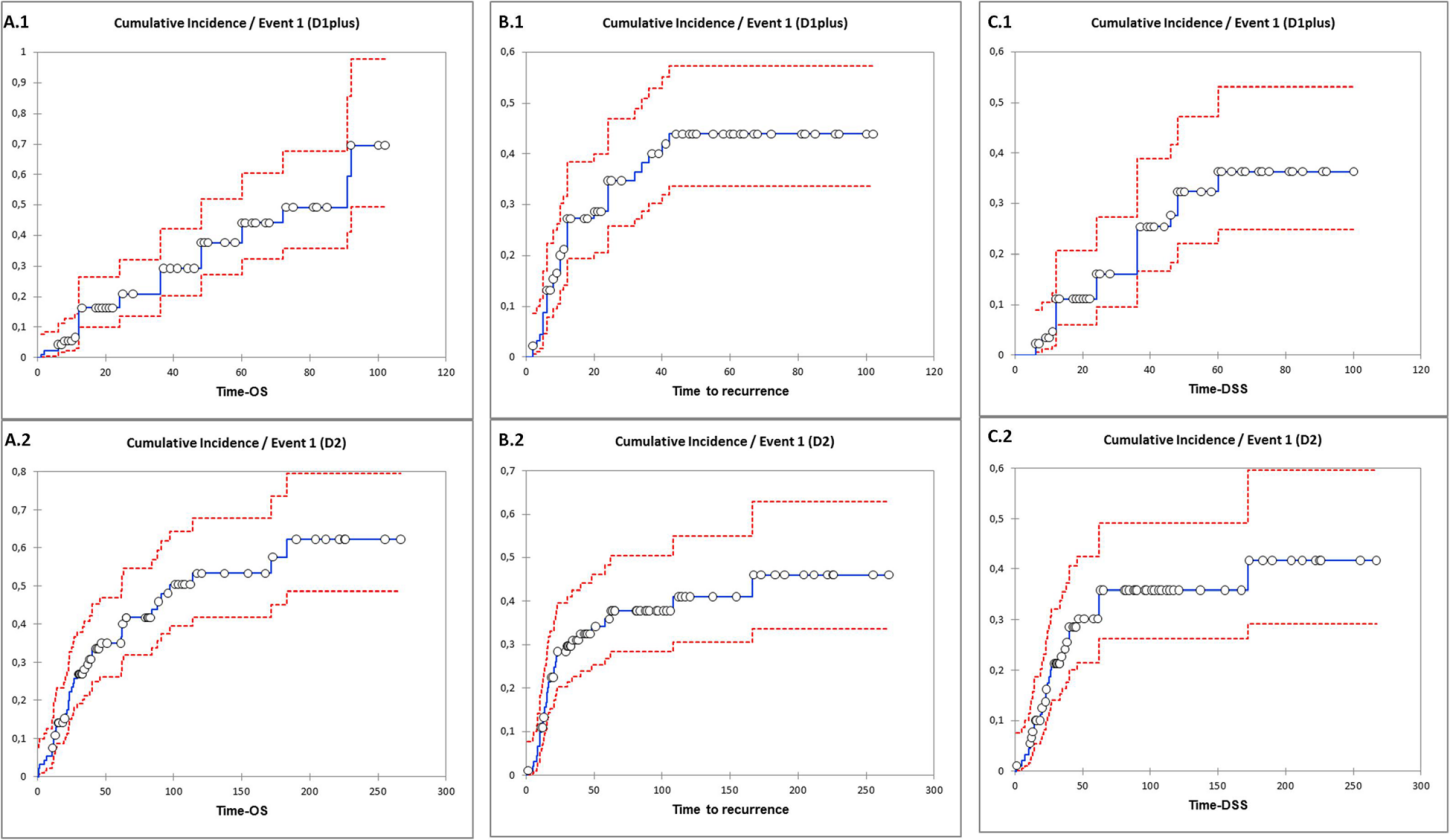
Cumulative incidence curves in D1-plus vs D2 nodal dissections; **A1.** Overall Survival (OS) in D1-plus and **A.2** in D2 nodal dissections; **B.1** Disease Free Survival (DFS) in D1-plus and **B.2** in D2 nodal dissections; **C.1** Disease Specific Survival (DSS) in D1-plus and **C.2** in D2 nodal dissections

The average follow-up in D1-plus group was 36.8 months, whereas in D2 group was 83.5 months, Table 1. Overall 67 relapses were observed, 34 in the D1-plus group and 33 in the D2 group of patients (p = NS). The cumulative incidence curves for OS, DFS and DSS events are reported in Fig. 4. Notably, curves were reported similar in the 2 cohorts of patients: OS D1-plus vs D2 Grey test *p* value 0.60; DFS D1-plus vs D2 Grey test *p* value 0.20; and DSS D1-plus vs D2, Grey test *p* value 0.98.

Cox regressions disclosed that total gastrectomies (HR 1.713, HR 95%CI 1.062–2.766), LNH < 15 (HR 3.010, HR 95%CI 1.658–5.467) and advanced stages (HR 3.675, HR 95%CI 2.218–6.090) correlated with worse OS. On the same extent total gastrectomies (HR 1.799, HR 95%CI 1.096–2.952), advanced stages (HR 5.958, HR 95%CI 3.419–10.383) and a D1-plus nodal dissection (HR 2.102, HR 95%CI 1.260–3.506) independently correlated with DFS, whereas total gastrectomies (HR 1.832, HR 95%CI 1.035–3.242), and advanced stages (HR 5.445, HR 95%CI 2.852–10.398) were correlated with a worse DSS, Table 2.

**Table 2:** Cox proportional hazard model

	Value	SE	Wald Chi-Square	Pr > Chi^2^	HR	HR Lower bound (95%)	HR Upper bound (95%)
Endopoint: OS							
Surgical Procedure	0.539	0.244	4.869	0.027	1.713	1.062	2.766
LNH < 15	1.102	0.304	13.113	0.000	3.010	1.658	5.467
Stage	1.302	0.258	25.530	< 0.0001	3.675	2.218	6.090
Endopoint: DFS							
Surgical Procedure	0.587	0.253	5.400	0.020	1.799	1.096	2.952
Stage	1.785	0.283	39.667	< 0.0001	5.958	3.419	10.383
Nodal Dissection	0.743	0.261	8.096	0.004	2.102	1.260	3.506
Endpoint: DSS							
Surgical Procedure	0.605	0.291	4.317	0.038	1.832	1.035	3.242
Stage	1.695	0.330	26.372	< 0.0001	5.445	2.852	10.398

### Literature review

Eighteen manuscripts were retrieved and reviewed, however 17 were excluded due to different outcome measures, or missing data of interest (Supplement File 1, exclusion list). Manual search provided two additional manuscripts and the three papers [23–25] were included for statistical computation along with the present case series.

**Fig. 5:**
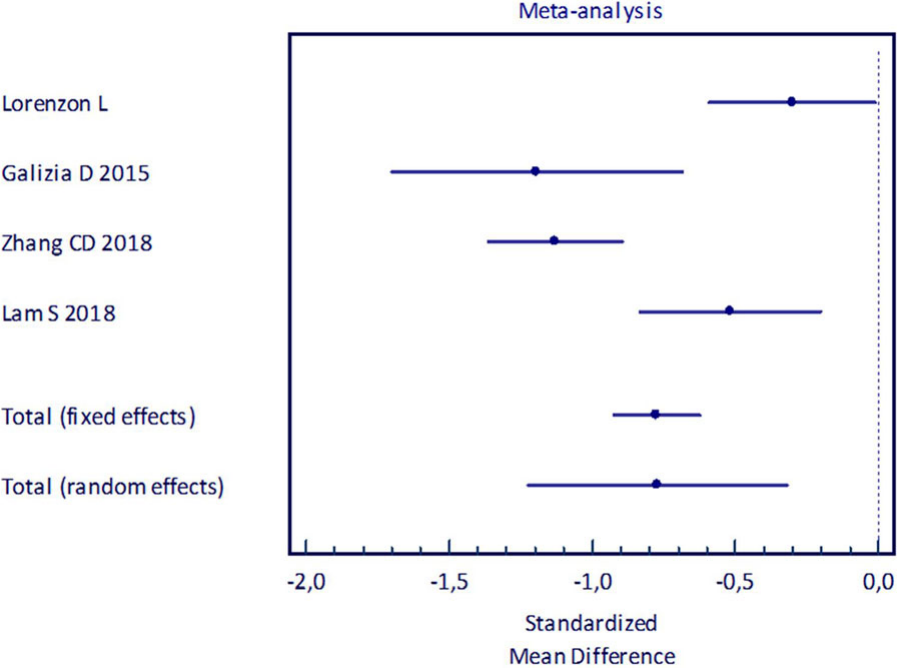
Forrest Plot of studies comparing LNH in D1-plus vs D2 lymphadenectomies

Survival outcome analysis was not possible due to missing data, thus the LNH was the sole outcome measure analyzed. Overall, 297 patients were analyzed in the D1-plus group (mean number of patients/study: 74.2 ± 35.6), whereas D2 sub-group included 556 patients (mean number of patients/study: 139.0 ± 102.3), Table 3. Mean LNH ranged from 7.9 ± 6.8 to 27.2 ± 12.5 in the D1-plus group. Opposite mean LNH ranged from 17.6 ± 9.2 to 31.2 ± 13.9 in the D2 group. LNH was documented highly in favor of D2 nodal dissection (SMD -0.772, 95%CI − 1.222 to − 0.322), Fig. 5.

**Table 3:** Metanalysis of studies comparing nodal harvest in D1-plus vs D2 nodal dissections

Study	D1-plus Number of patients	D2 Number of patients	Total Number of patients	SMD	95% CI	
*Lorenzon L*	93	93	186	−0.301	−0.592	−0.010
*Galizia D* 2015	36	37	73	−1.193	−1.702	−0.684
*Zhang CD* 2018	114	276	390	−1.130	−1.363	−0.897
*Lam S* 2018	54	150	204	−0.518	−0.835	− 0.201
Total (fixed effects)	297	556	853	−0.775	− 0.925	− 0.625
Total (random effects)	297	556	853	−0.772	−1.222	−0.322

Test for heterogeneity Q = 24.4933. DF = 3. *P* < 0.0001

## Discussion

Lymphadenectomy plays a key role in the surgical strategy of gastric cancer, mostly because nodal metastases could occur also in early stages of disease [26]. However, the extent of dissection remains an issue, because of the long-term diatribe between the Eastern countries supporting D2 lymphadenectomy and Western studies disclosing controversial results.

D1-plus approach is less frequently performed, and it has the aim of standing equidistant from these two opposite views in order to achieve a sufficient nodal harvest and allocate patients in appropriate pathological stages. Accordingly, a D1-plus dissection has been described as feasible and oncologically safe also for a laparoscopic approach [18]. An international cross-sectional survey conducted on 248 members of the International Gastric Cancer Association (IGCA) recently revealed that D1-plus nodal dissection was preferred in early gastric cancers (52% for distal and 54% for total gastrectomies) [27].

Just three very recent papers other than the present series could provide data for D1-plus vs D2 statistical comparison [23–25] and currently, an ongoing Korean clinical trial aims to compare D1-plus vs D2 in stage IB and II [19]. Zhang and co-authors documented a significant difference in the long-term OS (35.7% for D1+, 48.2% for D2), especially in nodal negative patients (36.6% for pN0-D1+ vs 63.9% for pN0-D2); consistently, lymphadenectomy was an independent prognostic factor of survival [24]. Similarly, Lam and associates documented advanced tumor stage (stages III and IV), D1/D1+ lymphadenectomy and postoperative morbidity were independent predictors of poor overall survival [25]. On the other hand, Galizia in another report, did not documented a survival benefit for D2 procedures, although, in this series, the resection included also splenectomy [23].

Results from the present analysis disclosed that LNH was in favor of D2 dissection, although LNH distributions and the rate of patients with less than 15 nodes harvested were similar, when comparing the two groups. Other experiences from the Eastern countries conducted on early gastric cancers reported optimal harvest also for D1-plus lymphadenectomy, up to a mean of 38.0 ± 16 nodes; results were, however, not computable in the analysis because a lack of a D2 comparison group [28].

Although we documented that D1-plus dissection could provide a sufficient harvest of more than 15 lymph nodes [6], the odds of under-staging the disease could be of concern, and the impact of such approach on survival should be further investigated in prospective trials.

Indeed, in the matched series, the cumulative events of OS, DFS and DSS and the related curves were similar, but Cox analyses disclosed that a D1-plus dissection could correlated with a worse DFS survival with HR 2.102.

Limitations of this study include the retrospective design and the differences in follow-up between the matched groups. However, D1-plus patients had a mean follow-up of about 3 years and usually relapses occur within this time-frame [29]. With respect of literature review, it was deemed necessary to include the present series in statistical analysis because of the scant number of studies retrieved. However, the adjunction of un-published series in a systematic review rarely could impact its results, but on the contrary may be important in case of few relevant studies in the field [30, 31].

## Conclusion

In conclusion, D2 provided greater LNH than D1-plus dissections. Further prospective studies will help in defining the benefits and limitations of nodal harvest extent also with respect to the peri-operative outcome, morbidity and long term results.

## Supplementary information

**Additional file 1: Figure S1.** Propensity Score Method. Unmatched vs Matched cohorts distribution.

**Additional file 2:Supplement File 1.** Exclusion List

## Data Availability

Not applicable.

## Abbreviations

LNH: Lymph-node harvest
LNR: Lymph-node ratio
OS: Overall survival
DFS: Disease free survival
DSS: Disease specific survival
SMD: Standardized mean difference
