# Supplementary figures and images for: D1-plus vs D2 nodal dissection in gastric cancer: a propensity score matched comparison and review of published literature
Source: BMC Surg. 2020 Jun 10;20:126. doi: 10.1186/s12893-020-00714-x (PMC7285465; doi:10.1186/s12893-020-00714-x)

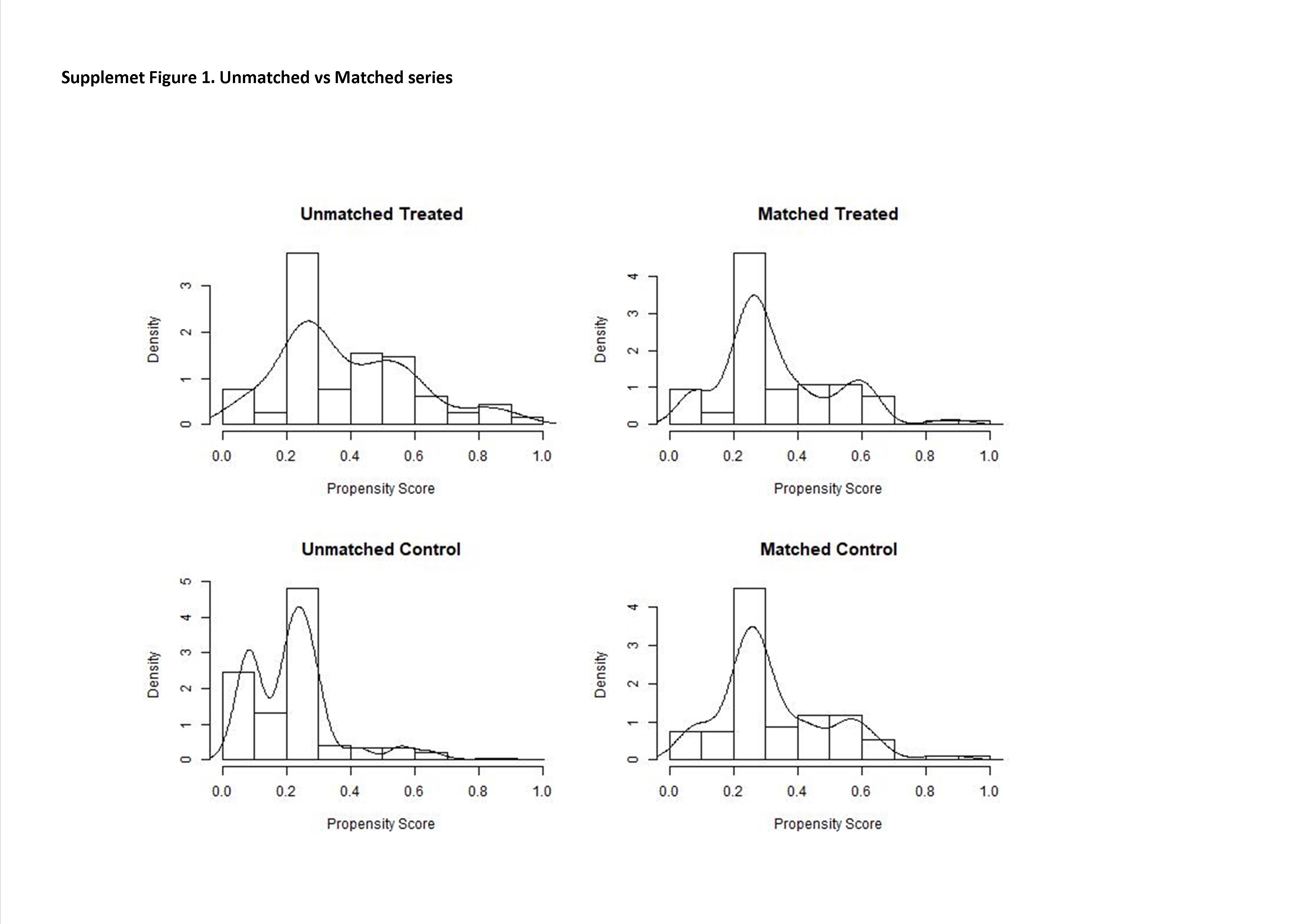

Supplement: Supplementary file 1 — Additional file 1: Figure S1. Propensity Score Method. Unmatched vs Matched cohorts distribution. [file 12893_2020_714_MOESM1_ESM.jpg]
