# Supplementary material for: D1-plus vs D2 nodal dissection in gastric cancer: a propensity score matched comparison and review of published literature
Source: BMC Surg. 2020 Jun 10;20:126. doi: 10.1186/s12893-020-00714-x (PMC7285465; doi:10.1186/s12893-020-00714-x)
Supplement: Supplementary file 2 — Additional file 2:Supplement File 1. Exclusion List [file 12893_2020_714_MOESM2_ESM.docx]

**Supplement File 1. Exclusion List**

| N | Study | Exclusion |
| --- | --- | --- |
| 1 | Hiki N, Katai H, Mizusawa J, Nakamura K, Nakamori M, Yoshikawa T, Kojima K, Imamoto H, Ninomiya M, Kitano S, Terashima M; Stomach Cancer Study Group of Japan Clinical Oncology Group. Long-term outcomes of laparoscopy-assisted distal gastrectomy with suprapancreatic nodal dissection for clinical stage I gastric cancer: a multicenter phase II trial (JCOG0703).*Gastric Cancer* 2018;**21**:155-161. | No D2 patients |
| 2 | Brenkman HJ, Haverkamp L, Ruurda JP, van Hillegersberg R. Worldwide practice in gastric cancer surgery. *World J Gastroenterol* 2016;**22**:4041-8. | Survey |
| 3 | Fujitani K, Yang HK, Mizusawa J, Kim YW, Terashima M, Han SU, Iwasaki Y, Hyung WJ, Takagane A, Park DJ, Yoshikawa T, Hahn S, Nakamura K, Park CH, Kurokawa Y, Bang YJ, Park BJ, Sasako M, Tsujinaka T; REGATTA study investigators. Gastrectomy plus chemotherapy versus chemotherapy alone for advanced gastric cancer with a single non-curable factor (REGATTA): a phase 3, randomised controlled trial. *Lancet Oncol* 2016;**17**:309-18. | D1 Lymphadenectomy |
| 4 | de Steur WO, Hartgrink HH, Dikken JL, Putter H, van de Velde CJ. Quality control of lymph node dissection in the Dutch Gastric Cancer Trial. *Br J Surg* 2015;**102**:1388-93. | D1 Lymphadenectomy |
| 5 | Talaiezadeh AH, Asgari M, Zargar MA. Mortality and Morbidity and Disease Free Survival after D1 and D2 Gastrectomy for Stomach Adenocarcinomas. *Asian Pac J Cancer Prev* 2015;**16**:5253-6. | D1 Lymphadenectomy |
| 6 | Mukherjee S, Hurt CN, Gwynne S, Bateman A, Gollins S, Radhakrishna G, Hawkins M, Canham J, Lewis W, Grabsch HI, Sharma RA, Wade W, Maggs R, Tranter B, Roberts A, Sebag-Montefiore D, Maughan T, Griffiths G, Crosby T. NEOSCOPE: a randomised Phase II study of induction chemotherapy followed by either oxaliplatin/capecitabine or paclitaxel/carboplatin based chemoradiation as pre-operative regimen for resectable oesophageal adenocarcinoma. **BMC Cancer** 2015;**15**:48. | Siewert Type 1-2 gastro-oesophageal junction |
| 7 | Aoyama T, Fujikawa H, Cho H, Ogata T, Shirai J, Hayashi T, Rino Y, Masuda M, Oba MS, Morita S, Yoshikawa T. A methylene blue-assisted technique for harvesting lymph nodes after radical surgery for gastric cancer: a prospective, randomized, controlled study. *Am J Surg Pathol* 2015;**39**:266-73. | No LNH data |
| 8 | Deng J, Zhang R, Pan Y, Wang B, Wu L, Jiao X, Bao T, Hao X, Liang H. Comparison of the staging of regional lymph nodes using the sixth and seventh editions of the tumor-node-metastasis (TNM) classification system for the evaluation of overall survival in gastric cancer patients: findings of a case-control analysis involving a single institution in China. *Surgery* 2014;**156**: 64-74. | D1 Lymphadenectomy |
| 9 | Kim HI, Hur H, Kim YN, Lee HJ, Kim MC, Han SU, Hyung WJ. Standardization of D2 lymphadenectomy and surgical quality control (KLASS-02-QC): a prospective, observational, multicenter study [NCT01283893]. *BMC Cancer* 2014;**14**:209. | Trial protocol |
| 10 | Glehen O, Passot G, Villeneuve L, Vaudoyer D, Bin-Dorel S, Boschetti G, Piaton E, Garofalo A. GASTRICHIP: D2 resection and hyperthermic intraperitoneal chemotherapy in locally advanced gastric carcinoma: a randomized and multicenter phase III study. *BMC Cancer* 2014;**14**:183. | Trial protocol |
| 11 | Degiuli M, Sasako M, Ponti A, Vendrame A, Tomatis M, Mazza C, Borasi A, Capussotti L, Fronda G, Morino M; Italian Gastric Cancer Study Group. Randomized clinical trial comparing survival after D1 or D2 gastrectomy for gastric cancer. *Br J Surg* 2014;**101**:23-31. | Trial protocol |
| 12 | Schmidt B, Chang KK, Maduekwe UN, Look-Hong N, Rattner DW, Lauwers GY, Mullen JT, Yang HK, Yoon SS. D2 lymphadenectomy with surgical ex vivo dissection into node stations for gastric adenocarcinoma can be performed safely in Western patients and ensures optimal staging. *Ann Surg Oncol* 2013;**20**:2991-9. | D1 Lymphadenectomy |
| 13 | Wada N, Kurokawa Y, Takiguchi S, Takahashi T, Yamasaki M, Miyata H, Nakajima K, Mori M, Doki Y. Feasibility of laparoscopy-assisted total gastrectomy in patients with clinical stage I gastric cancer. *Gastric Cancer* 2014;**17**:137-40. | No LNH data |
| 14 | Yano K, Nimura H, Mitsumori N, Takahashi N, Kashiwagi H, Yanaga K. The efficiency of micrometastasis by sentinel node navigation surgery using indocyanine green and infrared ray laparoscopy system for gastric cancer. *Gastric Cancer* 2012;**15**:287-91. | No LNH data |
| 15 | Katai H, Sasako M, Fukuda H, Nakamura K, Hiki N, Saka M, Yamaue H, Yoshikawa T, Kojima K; JCOG Gastric Cancer Surgical Study Group. Safety and feasibility of laparoscopy-assisted distal gastrectomy with suprapancreatic nodal dissection for clinical stage I gastric cancer: a multicenter phase II trial (JCOG 0703). *Gastric Cancer* 2010;**13**:238-44. | No LNH data |
| 16 | Feng XY, Li JH, Li JZ, Han ZX, Xing RD. Serum SCCA, Cyfra 21-1, EGFR and Cyclin D1 levels in patients with oral squamous cell carcinoma. *Int J Biol Markers* 2010;**25**:93-8 | Oral squamous cell carcinoma |
| 17 | Songun I, Putter H, Kranenbarg EM, Sasako M, van de Velde CJ. Surgical treatment of gastric cancer: 15-year follow-up results of the randomised nationwide Dutch D1D2 trial. *Lancet Oncol* 2010;**11**:439-49. | D1 Lymphadenectomy |
